# Periodontal Lesions among Maxillary Sinus Mucosal Thickening Visiting the Department of Oral Medicine and Radiology of a Tertiary Care Centre

**DOI:** 10.31729/jnma.8454

**Published:** 2024-02-29

**Authors:** Harleen Bali, Manisha Neupane, Swagat Kumar Mahanta, Deepa Niroula, Chandan Upadhyaya, Dashrath Kafle

**Affiliations:** 1 Department of Oral Medicine and Radiology, Kathmandu University School of Medical Sciences, Dhulikhel, Kavrepalanchok, Nepal; 2 Dental Department, Seti Provincial Hospital, Dhangadi, Kailali, Nepal; 3 Department of Communityand Public Health Dentistry, Kathmandu University School of Medical Science, Dhulikhel, Kavrepalanchok, Nepal; 4 Department Oral Medicine and Radiology, Institute of Medicine, Maharajganj, Kathmandu, Nepal; 5 Department of Oral and Maxillofacial Surgery, Kathmandu University School of Medical Sciences, Dhulikhel, Kavrepalanchok, Nepal; 6 Department of Orthodontics, Kathmandu University School of Medical Sciences, Dhulikhel, Kavrepalanchok, Nepal

**Keywords:** *cone beam computed tomography*, *maxillary sinus*, *periodontal disease*, *prevalence*

## Abstract

**Introduction:**

Maxillary sinus due to its proximity to posterior maxillary teeth could be affected by their pathology. Since cone beam computed tomography is the go-to for 3-D imaging in dental set-up. This study aimed to find out the prevalence of periodontal lesions among maxillary sinus mucosal thickening visiting the Department of Oral Medicine and Radiology of a tertiary care centre.

**Methods:**

This descriptive cross-sectional study was conducted in the Department of Oral Medicine and Radiology of a tertiary care centre from 01 February 2021 to 30 June 2021 after obtaining ethical approval from the Institutional Review Committee. The medical records from 01 January 2019 to 31 December 2019 were retrieved. The identification of maxillary sinus mucosal thickening of more than 3 mm, in cone beam computed tomography images, was registered separately for the right and left sinuses of each patient. The presence of periodontal lesions of posterior teeth was recorded. A convenience sampling method was used. The point estimate was calculated at a 95% Confidence Interval.

**Results:**

Among 195 maxillary sinus mucosal thickening, periodontal lesions were found in 74 (37.95%) (31.14-44.76, 95% Confidence Interval) maxillary sinuses of 46 patients. The mean age was 53.67±12.72 years and 30 (65.21%) were males.

**Conclusions:**

The prevalence of periodontal lesions among maxillary sinus mucosal thickening in CBCT images was similar to other studies done in similar settings.

## INTRODUCTION

Maxillary sinus due to its proximity to teeth and its surrounding structure forms an inevitable part of dentistry. Maxillary sinusitis of odontogenic origin, which was reported by Maloney in 1968 for the first time, has since become a topic of research.^1^ Recent efforts have shown that most instances of maxillary sinusitis can be traced to odontogenic origins.^2^

Since conventional diagnostics (i.e. intraoral and panoramic radiographs) in the past showed limited reliability, cone-beam computed tomography (CBCT) is now widely used for imaging studies of the oral cavity and the maxillofacial region. In comparison with conventional diagnostics, CBCT provides superior diagnostic accuracy in defining periodontal bone defects and the soft-tissue morphology of the maxillary sinus floor.^3^

This study aimed to find out the prevalence of periodontal lesions among patients with maxillary sinus mucosal thickening visiting the Department of Oral Medicine and Radiology of a tertiary care centre.

## METHODS

This descriptive cross-sectional study was done in the Department of Oral Medicine and Radiology, Kathmandu University School of Medical Sciences, Dhulikhel, Kavrepalanchok, Nepal from 01 February 2021 to 30 June 2021. The medical records from 01 January 2019 to 31 December 2019 were selected for the present study. Ethical approval was obtained from the Institutional Review Committee (Reference number: 40/20). Patients above 18 years of age with CBCT images showing the entire maxillary sinuses bilaterally or at least the four sinus walls completely with premolars and molars, at least one of them with contact with the floor of the maxillary sinus and the thickness of more than 3 mm were included.^4^ Images of low-resolution quality, distorted, overlapped, unclear images and/or those in which the presence of metallic artefacts impaired sinus visualization and patients with maxillary sinus trauma, edentulous patients, that is, missing posterior maxillary teeth were excluded. A convenience sampling method was used and sample size was calculated using the following formula.


$$\begin{array}{ll} \text{n} & ={\text{Z}}^{2}\times \frac{\text{p}\times \text{q}}{{\text{e}}^{\text{2}}}\\ & =1.96^{2}\times \frac{0.05\times 0.05}{0.08^{2}}\\ & =151 \end{array}$$

Where,

n = minimum required sample size
Z = 1.96 at a 95% confidence interval
p = prevalence taken as 50% for maximum sample size calculation
q = 1-p
e = margin of error, 8%

The minimum sample size calculated was 151. However, we have included a total of 195 thickened maxillary sinuses.

CBCT images were carefully examined for coronal, axial and sagittal views. All radiographs were taken using a Dentium Rainbow CBCT machine (having specifications such as scan time: 20 seconds, peak voltage: 100 kVp, tube current: 12 mA, Field of View: 16 × 18 cm, voxel size: 300 μm). All CBCTs were shot at 80 kVp, 7.0 mA and a scan time of 17 seconds in standard mode. Volume CT data was acquired. Multi-planar reconstruction was performed on a viewing workstation to obtain axial, saggital and coronal images. The obtained images were viewed and analyzed in Rainbow TM ImageViewer Version 1.0.0.0. The images were viewed on the same computer screen using the same image viewer, under ambient light with all curtains closed by an oral radiologist with more than three years of experience in CBCT reporting.

The identification of thickness was registered separately for the right and left sinuses of each patient. The presence of the first and second premolar, first, second, and third molar teeth in both the right and left maxillary posterior regions was recorded. The presence of periodontal lesions (alveolar bone loss, combined lesions) of these teeth was also recorded.

Periodontitis was identified as alveolar bone resorption. The alveolar crest is the point at which the periodontal ligament maintained a normal width. The normal height of the alveolar crest was defined as 2 mm apical to the cement-enamel junction (CEJ).^4^ Periodontal bone loss was classified as severe when ≥1 side of a tooth below the sinus had bone loss >50% of the total root length. Periodontal bone loss was classified as moderate when ≥1 side of a tooth below the sinus had a bone loss of 25% to 50%. Periodontal bone loss was classified as mild when all teeth below the sinus had bone loss <25%.^5^ Also noted were teeth with periodontitis associated with endodontic disease i.e. endodontic-periodontal lesion, periodontal-endodontic lesion, combined lesion.^6^

The data obtained was recorded in Microsoft Excel 2019 and analyzed using the IBM SPSS Statistics version 20.0.

## RESULTS

Among 195 maxillary sinuses with mucosal thickening (143 patients), periodontal lesions were found in 74 (37.95%) (31.14-44.76, 95% CI) maxillary sinuses of 46 patients. The age range of participants was 30-76 years with a mean age of 53.67±12.72 years. A total of 30 (65.21%) patients were males.

**Table 1:** Alveolar bone loss severity in periodontally involved teeth about the maxillary sinuses (n = 74).

Alveolar bone n (%)	Loss (%)	Right (n = 41) n (%)	Left (n = 33) n (%)
<25	10 (13.51)	6 (14.63)	4 (12.12)
25-50	44 (59.46)	23 (56.09)	21(63.63)
>50	20 (27.03)	12 (29.26)	8 (24.24)

## DISCUSSION

In our study, the prevalence of periodontal lesions was found in 37.95% of maxillary sinuses with mucosal thickness of more than 3 mm. Similarly, periodontal bone loss was seen in 30 % of the patients in a study done in Turkey.^7^ In a study among 221 periodontal patients, 103 (48.9%) displayed Maxillary Thickness.^8^ A study done in Brazil reported that patients with periodontal disease in posterior maxillary teeth showed a 3.45-fold higher risk of developing maxillary sinusitis.^7^ Data from some studies showed odds ratio values between 2.5 and 31.8 for mucosal thickening concerning various degrees of periodontal disease.^9^

Maxillary sinus due to its approximation to dental structures makes it inevitable to be studied by dentists whether in terms of diagnosis or management. The respiratory epithelium lining the paranasal sinus's mucosa is 1 mm thick normally. An increase of 10 to 15-fold in the sinus mucosa thickness can be seen in times of inflammation.^4^

In this study more number of patients with periodontal disease were males and the mean age of patients was 53.67 years similar to a study done in China in 2021 which reported 56.15% males with a mean age of 54.1 years.^10^ In our study, a higher prevalence of moderate bone loss was noted in around 60%. Taiwan 2020 study, showed that severe periodontal bone loss was significantly associated with mucosal thickening.^11^ It has been observed that the thickness of sinus mucosa at sites in which a tooth was extracted following periodontal disease was higher than those extracted due to pulpal disease and root fracture, supporting the role of periodontal disease on mucosal thickening of the maxillary sinus.^12^ In contrast, a study done in Switzerland reported that periodontal lesions and periapical lesions were not associated with sinus mucosal thickening.^3^ But then their study included not just mucosal thickening but mucosal cysts, mucoceles, and others when performing regression analysis.

Studies have shown that proximity of the lesion to the maxillary sinus may lead to its spread to the later and evoke a response. The anaerobic Prevotella species, Fusobacterium species, and Peptostreptococcus species found in periodontal disease have been cultured from sinus infections with secondary odontogenic cause.^13,14^ These products can reach the sinus mucosa through direct diffusion through the porous maxillary bone, causing maxillary sinusitis.^15^ Thus periodontium pathology needs to be treated when planning any treatment for maxillary sinusitis or implant placement in the region.

The present study being retrospective could not take into account the history, symptoms and signs concerning maxillary sinus as well as pathology of odontogenic origin. The study is representative of a particular geographic region. Therefore the authors advise further studies to be conducted in larger sample sizes to overcome the aforementioned limitations and also to validate and generalize the results. Since the cause for maxillary sinus mucosal thickening can be various, a prospective cohort study design is highly recommended.

## CONCLUSIONS

The prevalence of periodontal lesions among maxillary sinus mucosal thickening in CBCT images was similar to other studies done in similar settings. Further studies with larger sample sizes and prospective cohort designs are recommended to validate and generalize results, while interdisciplinary collaboration between dental and medical professionals is essential for a comprehensive understanding of the multifactorial aspects influencing maxillary sinus conditions associated with periodontal disease.
